# Reporting of clinical trials: a review of research funders' guidelines

**DOI:** 10.1186/1745-6215-9-66

**Published:** 2008-11-25

**Authors:** Kerry Dwan, Carrol Gamble, Paula R Williamson, Douglas G Altman

**Affiliations:** 1 Centre for Medical Statistics and Health Evaluation, University of Liverpool, Liverpool, UK; 2 Centre for Statistics in Medicine, University of Oxford, Oxford, UK

## Abstract

**Background:**

Randomised controlled trials (RCTs) represent the gold standard methodological design to evaluate the effectiveness of an intervention in humans but they are subject to bias, including study publication bias and outcome reporting bias. National and international organisations and charities give recommendations for good research practice in relation to RCTs but to date no review of these guidelines has been undertaken with respect to reporting bias.

**Methods:**

National and international organisations and UK based charities listed on the Association for Medical Research Charities website were contacted in 2007; they were considered eligible for this review if they funded RCTs. Guidelines were obtained and assessed in relation to what was written about trial registration, protocol adherence and trial publication. It was also noted whether any monitoring against these guidelines was undertaken. This information was necessary to discover how much guidance researchers are given on the publication of results, in order to prevent study publication bias and outcome reporting bias.

**Results:**

Seventeen organisations and 56 charities were eligible of 140 surveyed for this review, although there was no response from 12. Trial registration, protocol adherence, trial publication and monitoring against the guidelines were often explicitly discussed or implicitly referred too. However, only eleven of these organisations or charities mentioned the publication of negative as well as positive outcomes and just three of the organisations specifically stated that the statistical analysis plan should be strictly adhered to and all changes should be reported.

**Conclusion:**

Our review indicates that there is a need to provide more detailed guidance for those conducting and reporting clinical trials to help prevent the selective reporting of results. Statements found in the guidelines generally refer to publication bias rather than outcome reporting bias. Current guidelines need to be updated and include the statement that all primary and secondary outcomes prespecified in the protocol should be fully reported and should not be selected for inclusion in the final report based on their results.

## Introduction

Randomised controlled trials (RCTs) are planned experiments, involving the random assignment of patients to interventions, and are seen as the gold standard of methodological designs to evaluate the effectiveness of a treatment in medical research in humans [1].

However, empirical research consistently suggests that published work is more likely to be statistically significant (P < 0.05) than unpublished research, and significant results have a higher probability of being published than non-significant outcomes [2]. Evidence also suggests that published research without statistically significant results takes much longer to achieve publication than its statistically significant counterparts, further biasing evidence over time [3-5]. Such "study publication bias" is well recognised as a potential threat to the validity of any inference made as a result of a review of the literature [6].

Outcome reporting bias (ORB) has been defined as the selection on the basis of the results of a subset of the original variables recorded for inclusion in publication of trials [7]. Recent work [8-14] provides direct empirical evidence for the existence of outcome reporting bias. Chan et al [11] compared publications with protocols for 102 RCTs and found that statistically significant outcomes had a higher odds of being fully reported compared to non-significant outcomes for data both on efficacy (pooled odds ratio 2.4, 95% confidence interval 1.4 to 4.0) and safety (4.7, 1.8 to 12). Further, 62% of trials had at least one primary outcome that was changed, introduced, or omitted. A second study of 48 trials funded by the Canadian Institutes of Health Research found similar results [10] as have more recent studies [12,13]. Other researchers have also concluded the problem to be major, and deserving of substantially more attention than it currently receives [15].

The registration of RCTs before initiation, on clinical trial registries [16], is one step forward in trying to prevent reporting biases as there is documentation that the trials exist, so that reviewers are able to search these registries in order to locate unpublished trials to include in a systematic review.

Organisations that fund clinical trials often issue research guidelines to aid trialists in the conduct of their study. A review of these guidelines has been conducted to discover how much guidance research funders give concerning the publication of results. That review is the focus of this report.

Particular international guidelines [ICH Harmonised Tripartite Guideline for Good Clinical Practice (E6) [17], The EU Clinical Trials Directive [18], The Declaration of Helsinki [19] and the CONSORT Statement [20] were frequently referred to by funders of clinical trials and are thus briefly described first in terms of their coverage of publication issues.

The International Conference on Harmonisation Good Clinical Practice (ICH GCP) guideline came into effect in 1997 and "is an international ethical and scientific quality standard for designing, conducting, recording, and reporting trials that involve the participation of human subjects [17]." It was introduced to provide a unified standard for the United States, EU, and Japan to facilitate acceptance of clinical data by the respective regulatory authorities [21]. The ICH GCP guidelines do not mention trial registration but do state that "All important deviations related to study inclusion or exclusion criteria, conduct of the trial, patient management or patient assessment should be described. Adherence to the study protocol is essential. If modification of the protocol becomes necessary a clear description of the rationale for the modification should be provided in a protocol amendment." This relates to the issue of ORB because if outcomes are changed from those stated in the protocol for valid reasons, this should be documented before the write up of any trial reports so that it is obvious that results have not been selectively reported based on their results.

Regarding the primary outcome of a trial, the ICH GCP guidelines state "The primary measurements and endpoints used to determine efficacy should be clearly specified. There should generally only be one primary variable. The primary variable should be specified in the protocol, along with the rationale for its selection. Redefinition of the primary variable after unblinding will almost always be unacceptable, since the biases this introduces are difficult to assess." The guidelines also state that "The results of a clinical trial should be analysed in accordance with the plan prospectively stated in the protocol and all deviations from the plan should be indicated in the study report. In the statistical section of the clinical study report the statistical methodology should be clearly described including when in the clinical trial process methodology decisions were made." This indicates that the primary outcome should usually not change between protocol and publication of the trial report; if there is a change, it should be documented and explained in the trial report.

The Declaration of Helsinki [19] was first adopted in 1964 and has since been revised five times by the World Medical Association, most recently in 2000, although clarifications have been added since (2002 and 2004). Publication is discussed, stating that "Both authors and publishers have ethical obligations. In publication of the results of research, the investigators are obliged to preserve the accuracy of the results. Negative as well as positive results should be published or otherwise publicly available. Sources of funding, institutional affiliations and any possible conflicts of interest should be declared in the publication. Reports of experimentation not in accordance with the principles laid down in this Declaration should not be accepted for publication."

The CONSORT (Consolidated Standards of Reporting Trials) Statement is intended to improve the reporting of a randomised controlled trial [20]. It comprises a checklist of 22 essential items to be reported and a flow diagram. CONSORT includes a recommendation that the methods section should include information on 'clearly defined primary and secondary outcome measures and, when applicable, any methods used to enhance the quality of measurements'. The results section of CONSORT states 'For each primary and secondary outcome, a summary of results for each group, and the estimated effect size and its precision (e.g. 95% confidence interval)' should be reported.

The EU Clinical Trials Directive 2001 (updated in 2005) [18] was transposed into UK law through The Medicines for Human Use (Clinical Trials) Regulations 2004 [22]. The aims of this Directive was to "protect the rights, safety and well being of trial participants, to simplify and harmonise the administrative provisions governing clinical trials and to establish a transparent procedure that would harmonise trial conduct in the EU and ensure the credibility of results." The Directive refers to the Declaration of Helsinki and discusses the monitoring of trials and protocol adherence, stating that "after the commencement of the clinical trial, the sponsor may make amendments to the protocol. If those amendments are substantial and are likely to have an impact on the safety of the trial subjects or to change the interpretation of the scientific documents in support of the conduct of the trial, or if they are otherwise significant, the sponsor shall notify the competent authorities of the Member State or Member States concerned of the reasons for, and content of, these amendments and shall inform the ethics committee or committees concerned." This relates to the issue of ORB due to the reasons stated above regarding protocol deviations/amendments for the ICH GCP guidelines.

## Methods

A survey of major research organisations and UK charities (see Additional file 1, Appendix 1), using the same list as The DAMOCLES project [23], was undertaken to examine whether existing guidelines for research conduct refer to issues related to reporting biases. UK based charities listed on the Association for Medical Research Charities website were contacted (see Additional file 1, Appendix 1). National and international organisations were selected if they funded research in the United Kingdom, since it was felt important to obtain information from different funding sources and from different international geographical locations. Research organisations were eligible if they fund clinical trials.

### Ascertainment

Search terms that were used when searching for guidelines on the websites of major research organisations included; "research practice", "research conduct", "registration of trial and outcomes", "protocol adherence", "protocol amendments", "reporting results", "publication" and "researchers' handbook". When guidelines were identified they were obtained and reviewed. The organisations were also contacted by email to ensure all of the information available was obtained.

For some organisations no document could be located through their website. In these cases we requested information by a shortened version of the questionnaire (see Additional file 1, Appendix 2) on issues related to outcome reporting. This questionnaire was sent via email if possible, or by post otherwise.

KD extracted information from all guidelines that were obtained, and PW carried out a random check that all relevant information had been extracted. The information relating to each organisation or charity was then sent to the contact individual for review so that we could ensure that all information was correct and if there was anything else to be added.

We also contacted all 108 charities (see Additional file 1, Appendix 1) listed on the Association for Medical Research Charities website  to find out if they fund clinical trials, and if so, what was the maximum grant available. A questionnaire (see Additional file 1, Appendix 2) was then sent to those charities that funded RCTs. The search of the AMRC website was done more than once.

The final search of the AMRC website in September 2007 indicated that 7 more charities had been added, while 2 from the original list that we contacted had been removed (National Eczema Society and Tyneside Leukaemia Research Association) and 2 charities have been incorporated into Epilepsy Research UK (Epilepsy Research Foundation and Fund for Epilepsy).

Data from all organisations and charities were entered into a Microsoft Access database with data entry screens in the form of the original questionnaire (see Additional file 1, Appendix 3). The questionnaire in appendix 2 (see Additional file 1) was used as a means of contacting the funding agency and requesting a copy of their guidelines and the questionnaire in appendix 3 (see Additional file 1) was used when reading through guidelines.

Under "protocol adherence/amendment" we considered whether there was any reference in the guidelines to these issues (i.e. do the guidelines state that the protocol should be adhered to? Do the guidelines give any information on what to do if there are changes to the protocol?) This would cover the issue of ORB as any legitimate changes to changes of outcomes should be reported. Under "trial publication" we sought any statement referring to publication (i.e. if it should be published, where it should be published, when it should be published, what should be included in the publication etc.). For "monitoring against guidelines" we looked for a reference to monitoring for adherence to the published guidelines.

### Analysis

Descriptive results from the questionnaire are presented. Data are tabulated and excerpts found in the guidelines relating to the issue of outcome reporting are used to support statements made.

## Results

### Organisations and charities

Twenty five organisations were contacted of which 17 were eligible. There were 115 charities listed on the AMRC website (108 originally listed charities including the 2 charities which were later removed from the website plus 7 newly added charities) of which 56 were eligible (Table 1). Eight organisations and 48 charities were not eligible as they did not fund clinical trials. There was no reply from the remaining 11 charities.

**Table 1:** Summary of response from organisations and charities

**Action**	**Number of organisations (percentage)**	**Number of charities (percentage)**	**Total (percentage)**
Contacted	25	115	140

			

**Eligibility**			

Eligible	17 (68%)	56 (49%)	73 (52%)

Not eligible	4 (16%)	48 (42%)	52 (37%)

No reply	0 (0%)	11 (9%)	11 (8%)

Issue guidelines but do not fund RCTs	4 (16%)	0 (0%)	4 (3%)

			

**Guidelines (n = 73)**			

Guidelines/terms and conditions received	14 (82%)	52 (93%)	66 (90%)

Limited contact	2 (12%)	1 (2%)	3 (4%)

No guidelines	1 (6%)	1 (2%)	2 (3%)

Could not send due to confidentiality	0 (0%)	2 (3%)	2 (3%)

Research funder not checked table of information	3 (18%)	8 (14%)	11 (15%)

			

**Not referred to other guidelines**	3 (18%)	14 (25%)	17 (23%)

**Referred to other guidelines:**^1^	14 (82%)	42 (75%)	56 (77%)

Association of Medical Research Charities	0 (0%)	18 (43%)	18 (32%)

Department of Health Research Governance Framework (England)	3 (21%)	15 (36%)	18 (32%)

Medical Research Council	5 (36%)	13 (31%)	18 (32%)

International Conference on Harmonisation (ICH)	10 (71%)	4 (10%)	14 (25%)

Medicines for Human use	6 (43%)	8 (19%)	14 (25%)

Institution	0 (0%)	11 (26%)	11 (20%)

Declaration of Helsinki	7 (50%)	2 (5%)	9 (16%)

CONSORT	6 (43%)	0 (0%)	6 (11%)

Charity specific	0 (0%)	4 (10%)	4 (7%)

National regulations and guidelines	1 (7%)	3 (7%)	4 (7%)

Legal	0 (0%)	4 (10%)	4 (7%)

Wellcome Trust	0 (0%)	3 (7%)	3 (5%)

			

**Reference in guidelines/through contact (n = 73) to:**			

Trial registration explicitly	12 (71%)	7 (13%)	19 (26%)

Trial registration implicitly	0 (0%)	29 (52%)	29 (40%)

Protocol adherence/amendment explicitly	12 (71%)	20 (36%)	32 (44%)

Protocol adherence/amendment implicitly	2 (12%)	24 (43%)	26 (36%)

Trial publication explicitly	14 (82%)	35 (63%)	49 (67%)

Trial publication implicitly	1 (6%)	13 (23%)	14 (19%)

Monitoring against guidelines explicitly	11 (65%)	36 (64%)	47 (64%)

Monitoring against guidelines implicitly	1 (6%)	11 (20%)	12 (16%)

Publication of negative studies explicitly	6 (35%)	5 (9%)	11 (15%)

Publication of negative studies implicitly	3 (18%)	24 (43%)	27 (37%)

Publication of negative outcomes explicitly	6 (35%)	5 (9%)	11 (15%)

Publication of negative outcomes implicitly	3 (18%)	24 (43%)	27 (37%)

**1**. Each charity/organisation may refer to one or more other guidelines

Limited contact meant that there was initial contact with the organisation/charity to confirm that they did fund clinical trials and the maximum grant available was, but then no further information was forthcoming.

Institution means the guidelines refer to the guidelines issued by the university or other institution were the trial is to be conducted from.

Charity specific means that the charity has specific guidelines for the particular disease/illness but not necessarily good research practice guidelines.

National regulations and guidelines are any other guidelines issued nationally e.g. for the UK, America e.t.c.

Legal guidelines means the guidelines refer to specific legal guidelines for clinical trials or the specific disease/illness being studied.

### Search for guidelines

Websites for all 73 eligible organisations and charities were accessed but the success in searching for guidelines was mixed. Some were relatively easy to find and well directed by the organisation (e.g. Medical Research Council), while others proved more difficult to find. The websites for Health Technology Assessment (HTA) and National Institute of Health (NIH), for example, provided a lot of information making it difficult to find the required guidelines.

Fifty two charities and 14 organisations had guidelines for RCTs or sent the terms and conditions of a grant. There was limited contact with one charity and two organisations (and there was no relevant information on their websites), one charity and one organisation said they had no guidelines and two charities would not send their guidelines/terms and conditions due to confidentiality. Eight of the charities and three of the organisations did not respond when we asked them to check the information we had extracted.

The guidelines were requested as per Appendix 2 (see Additional file 1). In some cases we were sent the terms and conditions of the grant and when we followed up to request research guidelines nothing more was available. The terms and conditions were mostly concerned with the legalities of the funding and did not contain information that we were looking for.

As the websites were searched several times and all organisations and charities were also contacted on several occasions, the information regarding how many guidelines were identified on the websites is not available. Even if information was found on the website, each charity and organisation was contacted to enquire whether there was any other information available. Most of the information was obtained through contact with the organisation/charity. Even if the questionnaire was returned the question on how the guidelines were issued to the researcher was often not answered; only 30 (41%) organisations/charities stated that the guidelines were sent by post/email (11 (37%)), supplied with the grant application (11 (37%)) or researchers were referred to their website (8 (26%)).

### Guidelines

Table 1 summarises the responses from the organisations and charities that fund RCTs. By explicit reference it is meant that the charities/organisations guidelines referred to one of the guideline domains (trial registration, protocol adherence, publication, or monitoring) within their guidelines. By implicit it is meant that the charities/organisations guidelines referred to other guidelines such as CONSORT, ICH E6 guidelines etc which referred to the guideline domains.

Of the guidelines issued by the organisations and charities, 19 (26%) stated explicitly that a trial should be registered although a further 29 (40%) referred to trial registration implicitly through their reference to other guidelines such as AMRC, MRC or the Department of Health Research Governance Framework. Protocol adherence and protocol amendment were discussed explicitly in 32 (44%) and implicitly in 26 (36%) of the guidelines although not specifically in relation to outcomes; they state only that the protocol should be followed, all major amendments need ethics committee approval (or similar) and minor amendments need to be recorded. Publication was explicitly referred to in 49 (67%) and implicitly in 14 (19%) of the guidelines/terms and conditions. They state that trials should be monitored with regards to the guidelines (47 (64%) explicitly and 12 (16%) implicitly). Publication of negative and positive findings were explicitly referred to in 11 (15%) and implicitly in 27 (37%) of the guidelines/terms and conditions. The large majority refer to other guidelines (Table 1).

### Association of Medical Research Charity guidelines

The AMRC guidelines on good research practice [24] refers to the Department of Health Research Governance Framework, MRC guidelines for good clinical practice, and Wellcome Trust guidelines and state that the institution should implement good research practice. The guidelines encourage publication in peer reviewed journals and dissemination of research.

### Statements specific to outcomes, ORB or publication bias

Table 2 includes statements found in the guidelines that are specific to outcomes, ORB or publication bias. Eleven (15%) of the 73 organisations or charities and non-funding organisations mention the importance of publication of negative as well as positive findings. Three (4%) of the organisations specifically state that the statistical analysis plan should be strictly adhered to and all changes should be reported and fully justified in the final report. NIAID state that there should generally only be one primary outcome and that this should be clearly defined in the protocol.

**Table 2:** Statements specific to outcomes, ORB or publication bias

**Organisation/Charity**	**Statements specific to outcomes, ORB or publication bias**
Chief Scientist Office of the Scottish Executive Health Department	There should be free access to information both on research being conducted and on the findings of the research – positive or negative-once these have been subjected to appropriate scientific review. When established, findings (including negative findings) are published in ways that allow critical review and dissemination to those who could benefit from them.

Department of Health (England)	When established, findings (including negative findings) are published in ways that allow critical review and dissemination to those who could benefit from them.

World Health Organisation	Procedures for communicating deviation from the original statistical plan (any deviation from the original statistical plan should be described and justified in the protocol and/or in the final report). The study protocol may include; in the case of a negative outcome, an assurance that the results will be made available, as appropriate, through publication or by reporting to the drug registration authority. Both authors and publishers have ethical obligations. In publication of the results of research, the investigators are obliged to preserve the accuracy of the results. Negative as well as positive results should be published or otherwise publicly available. Sources of funding, institutional affiliations and any possible conflicts of interest should be declared in the publication. Reports of experimentation not in accordance with the principles laid down in this Declaration should not be accepted for publication.

Medical Research Council (UK)	The analysis should follow a carefully written analysis plan. All outcome measures stated in the protocol should be fully analysed. It is equally unethical not to report results, or to exaggerate the importance of results for medical practice or policy. Trials should be submitted to a peer reviewed journal irrespective of the results of the trial. The council's mission to improve quality of life also carries with it the imperative to report results that are directly relevant; there can be no freedom not to do so. This is also required as one of the conditions of gaining ethical approval for studies using human participants. It is increasingly recognised that that many results of clinical studies are not published but are nevertheless significant in building up an overall picture of the efficacy of a particular diagnostic, therapeutic or preventive approach.

The National Health and Medical Research Council (Australia)	Procedures for reporting any deviation(s) from the original statistical plan (any deviation(s) from the original statistical plan should be described and justified in the protocol and/or in the final report, as appropriate). Disseminating and communicating results, whether favourable or unfavourable, in ways that permit scrutiny and contribute to public knowledge and understanding.

The National Institute for Allergies and Infectious Diseases (USA)	Outcome measures should be prioritized. Generally, there should be just one primary variable, with evidence that it will provide a clinically relevant, valid and reliable measure of the primary objective.

Health and Social Care Research and Development Office (Northern Ireland)	When established, findings (including negative findings) are published in ways that allow critical review and dissemination to those who could benefit from them. Other researchers have access to the data on which the findings are based.

Ataxia UK	Ataxia UK recognises that journals normally publish only positive findings, and that negative findings may nevertheless be of value. Ataxia UK therefore reserves the right to publish summaries of negative as well as positive findings on its website or elsewhere.

Chronic Granulomatous Disorder Research Trust	The Institution will ensure that publication of findings in peer reviewed journals is sought as soon as possible during, and after conclusion, of the Project even where results prove negative.

Cystic Fibrosis Trust	Grantholders are expected to seek publication of findings in peer reviewed journals as soon as possible during, and after conclusion, of the project even where results prove negative.

Multiple Sclerosis Society of Great Britain and Northern Ireland	The Multiple Sclerosis Society requires its researchers to disseminate the results of the research that it funds in the usual manner, for example by publication in peer reviewed journals and presentation at meetings, as soon as possible during, and after conclusion, of the project even where results prove negative or inconclusive. Researchers should be honest in respect of their own actions in research and in their responses to the actions of other researchers. This applies to the whole range of research work, including experimental design, generating and analysing data, applying for funding, publishing results, and acknowledging the direct and indirect contribution of colleagues, collaborators and others.

Research into ageing	Unless otherwise stated in the Offer Letter, Grantholders are required by the Charity to seek publication of findings in peer reviewed journals (as appropriate) as soon as possible during, and after conclusion, of the Project even where results prove negative.

Tables 3 (see Additional file 2) and 4 (see Additional file 3) include details of information found in the guidelines or through response to questionnaires for organisations and charities respectively.

## Discussion

This review provides a narrative summary of the guidelines that are available from major organisations across the world and UK charities that fund RCTs. We found that funders of clinical trials have not yet incorporated recent concerns about ORB into their recommendations for researchers. There is a need to provide detailed guidance for those conducting and reporting clinical trials to help prevent the selective reporting of outcomes.

In this review we focussed on guidelines for good research practice for clinical trials. The main limitation of this review is that the search of organisations was not exhaustive. We concentrated mainly on organisations from Europe, USA, Australia and South Africa, and only UK charities have been investigated. However, the ICH guidelines, discussed below, are international.

The majority of guidelines provided no references to the problem for several of the questions in the questionnaire (see Additional file 1, Appendix 3); these were mainly questions specifically regarding outcomes. This is likely to be due to the fact that awareness of the problem of ORB has been limited over the past decade. As we have found, the importance of this issue has not filtered through to these guidelines.

Many of the guidelines state that a trial should be registered before it begins. The purpose of registration is to inform other researchers of trials that are being conducted; the aims include reducing publication bias and the duplication of research [6].

Several guidelines that we obtained discuss the importance of adherence to the protocol and state that amendments to the protocol should be reported. This issue is related to ORB as outcomes that are specified as primary or secondary in the protocol should be written up as such in the final report. Insertion of new outcomes and deletion of old outcomes should not occur unless an official amendment to the protocol was made, and such changes should be reported in journal articles. The ICH E3 [25] guideline states that "if the protocol did not identify the primary variables, the study report should explain how these critical variables were selected (e.g., by reference to publications, guidelines or previous actions by regulatory authorities) and when they were identified (i.e., before or after the study was completed and unblinded)." The Medicines and Healthcare products Regulatory Agency (MHRA) state that "new questions based on the observed data may well emerge during the analysis. This additional work should be strictly distinguished in the report from the work which was planned in the protocol." No such reports were found in the cohort of 102 trials studied by Chan et al [11] despite over half having changed their primary outcomes in some way.

Publication is often mentioned in funders' guidelines, stating that work should be published in a peer reviewed journal or at least stating that it should be disseminated. The Association of the British Pharmaceutical Industry states that work should be published and refers to guidelines on good publication practice [26], which aim to ensure that clinical trials sponsored by pharmaceutical companies are published in a responsible and ethical manner.

Many RCTs are carefully monitored by trial steering committees and this requirement is stated in several of the guidelines/terms and conditions. Monitoring tends to focus on whether trialists are following correct procedures. Therefore, issues such as protocol adherence and trial registration should be noticed and highlighted if they are not done correctly. Once a trial has been completed, such oversight may diminish. Ensuring that trial results are published may also be a concern of the data monitoring committee [23].

These four issues – registration of a trial, protocol adherence/amendment, publication and monitoring against guidelines – are important with regard to ORB, as the more rigorous the guidelines are in these areas, adherence makes ORB less likely.

Statements found in the guidelines generally refer to publication of negative and positive findings which could be interpreted as referring to study publication bias (i.e. non-publication of whole studies) or ORB (selective non-publication of outcomes). The empirical evidence of publication bias was first seen in the early 1990's [2] and much work has now been done in the area of publication bias since [6]. By contrast, studies providing the empirical evidence for ORB were published more recently between 2002 and 2008 [2]. Some of the more detailed and most often referred to guidelines, such as ICH [17,27,25,28] and MRC Good Research Practice guidelines [29], were written before 2000, when ORB first began to be recognised [7]. However, the Department of Health's Research Governance Framework [30] was updated in 2005 and provides no guidelines aimed specifically at preventing ORB.

Organisations tended to have their own guidelines whereas the charities mostly had terms and conditions but referred to some of the organisations' guidelines. This may reflect some of the charities being small and that some had only recently begun to fund RCTs.

The CONSORT Statement [20] addresses the write up of the final report and does not link this explicitly to the trial protocol. It does not specifically refer to ORB as it only states that outcome measures should be specified in the methods section and fully reported in the results section. Although this recommendation attempts to prevent partial reporting of outcomes (e.g. *p*-value only), and in addition may help identify outcomes that have not been reported, these recommendations alone will not prevent the selective reporting of outcomes. The CONSORT statement does not state that **all **outcomes that were included in the protocol should be stated in the methods section and reported in the results section of the final report. It also does not state that outcomes should not be upgraded or downgraded between the protocol and final report, or that new outcomes should not be added or old outcomes deleted based on their results. Therefore even when trialists follow the CONSORT Statement, it would be impossible to determine whether all outcomes in the protocol are reported in the final report. However, these concerns will be addressed in the revision of CONSORT that is under way. (DG Altman, personal communication)

Since this work has been completed, the members of the WHO Registry Platform Working Group on the Reporting of Findings of Clinical Trials have proposed that "the findings of all clinical trials must be made publicly available [31]."

In the USA legislation is in place so that legally researchers are required to make their findings publicly available within a specific timeframe [32]. The legislation states that "The expansion of the registry data bank to include results of clinical trials; beginning not later than 90 days after the date of the enactment of the Food and Drug Administration Amendments Act of 2007, for those clinical trials that form the primary basis of an efficacy claim or are conducted after the drug involved is approved or after the device involved is cleared or approved, the Secretary shall ensure that the registry data bank includes links to results information." It also includes specifics on the reporting of outcomes: "The primary and secondary outcome measures as submitted and a table of values for each of the primary and secondary outcome measures for each arm of the clinical trial, including the results of scientifically appropriate tests of the statistical significance of such outcome measures." It is hoped that these initiatives will reduce the problem of ORB.

This review forms part of the **Outcome Reporting Bias in Trials (ORBIT) project **[33]. It has been funded to investigate how prevalent outcome reporting bias is in trials within systematic reviews and whether this has a large impact on the conclusions of the reviews, as well as trying to understand why trialists do not report all outcomes and investigating the prevalence of ORB in individual patient data reviews on the Cochrane Collaboration Individual Patient Data Methods Working Group register. The identification of this gap in the guidelines is part of a suite of initiatives to reduce the problem of ORB.

## Conclusion

Our review indicates that there is a need to provide more detailed guidance for those conducting and reporting clinical trials to help prevent the selective reporting of outcomes. Current guidelines need to be updated, as many include statements regarding the publication of 'negative' studies to prevent publication bias but do not go as far as mentioning ORB. In the MRC guidelines the statement 'all outcomes stated in the protocol should be fully analysed' needs to be extended to specify that prespecified primary and secondary outcomes should be fully reported and that outcomes should not be selected for inclusion in the final report based on their results. Recommendations for text to include in research funders' guidelines are suggested (see Additional file 4).

The next step is to conduct a systematic review of empirical studies that have compared protocols to publications, to assess their adherence to specified guidelines. It would then be important to conduct a survey of trialists to see which guidelines they follow and their adherence to them, to assess their understanding of the issues discussed in this paper.

## Abbreviations

AMRC: Association for Medical Research Charities; CONSORT: Consolidated Standards of Reporting Trials; HTA: Health Technology Assessment; ICH GCP: The International Conference on Harmonisation Good Clinical Practice; MHRA: The Medicines and Healthcare products Regulatory Agency; MRC: Medical Research Council; NIAID: National Institute for Allergies and Infectious Diseases; NIH: National Institute for Health; ORB: Outcome Reporting Bias; RCT: Randomised Controlled Trial

## Competing interests

The authors declare that they have no competing interests.

## Authors' contributions

DGA conceived the idea for reviewing guidelines following the DAMOCLES project. All authors contributed to the design of the study.

KD contacted all organisations and charities, read through the guidelines obtained and drafted the manuscript. PRW did a random check of the guidelines to ensure all relevant details had been extracted and also provided comments on drafts of the manuscript. DGA and CG provided comments on all drafts of the manuscript.

## Supplementary Material

Additional file 1 Appendices for review of guidelines. A list of charities and organisations that were contacted and questionnaires that were completed. Click here for file

Additional file 2 Table 3 Summary of organisation guidelines. A table of findings for organisations that issue guidelines Click here for file

Additional file 3 Table 4 Summary of findings for charities that issue guidelines or terms and conditions. A table of findings for charities that issue guidelines Click here for file

Additional file 4 Text box 1: Recommendations for text to include in research funders' guidelines. Recommendations for text to include in research funders' guidelines. Click here for file
