# Supplementary material for: Reporting of clinical trials: a review of research funders' guidelines
Source: Trials. 2008 Nov 25;9:66. doi: 10.1186/1745-6215-9-66 (PMC2630961; doi:10.1186/1745-6215-9-66)
Supplement: Additional file 1 — Appendices for review of guidelines. A list of charities and organisations that were contacted and questionnaires that were completed. [file 1745-6215-9-66-S1.doc]

**Appendix 1**

**List of organisations approached**

Charities

Action Cancer

Action Medical Research

Action on Addiction

Alcohol & Education Research Council, The[[1]](#footnote-2)

Alzheimer's Research Trust

Alzheimer's Society

Arthritis Research Campaign

Association for International Cancer Research

Association for Spina Bifida and Hydrocephalus (ASBAH)

Asthma UK

Ataxia

Ataxia-Telangiectasia Society

BackCare

Bardhan Research and Education Trust of Rotherham^1^

Beit Memorial Fellowships for Medical Research^1^

Blackie Foundation Trust

Brain Research Trust

Breakthrough Breast Cancer

Breast Cancer Campaign

British Council for Prevention of Blindness

British Heart Foundation

British Liver Trust

British Lung Foundation

British Neurological Research Trust, The^1^

British Occupational Health Research Foundation

British Pain Society, The

British Retinitis Pigmentosa Society

British Sjögren's Syndrome Association

British Skin Foundation

BUPA Foundation

Cancer Research UK

CFS Research Foundation

Chest Heart and Stroke Scotland

Children with Leukaemia

Children's Liver Disease Foundation

Chronic Disease Research Foundation

Chronic Granulomatous Disorder Research Trust

Circulation Foundation (British Vascular Foundation)

CORE (Digestive Disorders Foundation)

Cystic Fibrosis Trust

Deafness Research UK (aka Hearing Research Trust)

Diabetes Research and Wellness Foundation

Diabetes UK

Dunhill Medical Trust^1^

Dystrophic Epidermolysis Bullosa Research Association (DEBRA)

EMF Biological Research Trust

Epilepsy Research Foundation[[2]](#footnote-3)

Fight for Sight (British Eye Research Foundation)

Foundation for Liver Research

Foundation for the Study of Infant Deaths

Fund for Epilepsy^2^

Guy's and St Thomas' Charity

The Healing Foundation

Heart Research UK

Huntington's Disease Association

Hypertension Trust

Inspire Foundation (aka Integrated Spinal Rehabilitation Foundation)

International Spinal Research Trust

Juvenile Diabetes Research Foundation UK

Kidney Research UK

[Kids Kidney Research](http://www.amrc.org.uk/HomePage/Default.aspx?Nav=479,536&member=13620)^1^

Lister Institute of Preventive Medicine

The Little Foundation

Ludwig Institute for Cancer Research

Marie Curie Research Institute

Mason Medical Research Foundation^1^

Medical Research Scotland (formally Scottish Hospital Endowments Research Trust, SHERT)

Meningitis Research Foundation

Meningitis Trust

Meningitis UK (Registered as Spencer Dayman Meningitis UK)

Migraine Trust

Motor Neurone Disease Association

Multiple Sclerosis Society of Great Britain and Northern Ireland

Muscular Dystrophy Campaign

National Association for Colitis & Crohn's Disease

National Eczema Society[[3]](#footnote-4)

National Eye Research Centre

National Osteoporosis Society

Neur[o-Disability Research Trust (formerly Living Again)](http://www.neuro-disability.org.uk/cat.asp?catid=20)

North West Cancer Research Fund

Northern Ireland Chest Heart & Stroke Association

Northern Ireland Leukaemia Research Fund

Novartis Foundation

Novo Nordisk UK Research Foundation

Nuffield Foundation

Parkinson's Disease Society of the UK

PBC Foundation

Primary Immunodeficiency Association

Progressive Supranuclear Palsy Association

Prostate Cancer Research Foundation, The

Psoriasis Association

Queen Victoria Hospital Blond McIndoe Research Foundation

RAFT (Restoration of Appearance & Function Trust)

Remedi

Research into Ageing

Roy Castle Lung Cancer Foundation, The

Royal College of Surgeons of England

Saint Peter's Trust for Kidney, Bladder & Prostate Research

Samantha Dickson Brain Tumour Trust

Sir Jules Thorn Charitable Trust

Society for Endocrinology

Sparks (Sport Aiding Medical Research for Kids)

Stroke Association

Tenovus

Tommy’s The Baby Charity

Tuberous Sclerosis Association

Tyneside Leukaemia Research Association^2^

Ulster Cancer Foundation

Wellbeing of Women

WellChild

Wellcome Trust

Wessex Medical Trust (aka Hope)

William Harvey Research Foundation

World Cancer Research Fund

Yorkshire Cancer Research

UK Governmental health departments

Chief Scientist Office of the Scottish Executive Health Department

Department of Health (England)

Health and Social Care Research and Development Office (Northern Ireland)

Health and Social Services Committee (Wales)

COREC

Industry

Association of the British Pharmaceutical Industry

International Conference on Harmonisation/International Federation of Pharmaceutical Manufacturers’ Associations

International

Community Research and Development Information Service (EU)

World Health Organisation

Regulatory

European Agency for the Evaluation of Medicinal Products

Food and Drug Administration (USA)

MHRA (UK)

The Canadian Agency for Drugs and Technologies in Health

Research and Development – governmental bodies and research councils

Canadian Institutes of Health Research

European Organisation for Research and Treatment of Cancer

Medical Research Council (UK)

Medical Research Council of South Africa (MRC-SA)

The Health Technology Assessment Programme (UK)

The National Cancer Institute (USA)

The National Health and Medical Research Council (Australia)

The National Heart, Lung and Blood Institute (USA)

The National Institute for Allergies and Infectious Diseases (USA)

The National Institute for Child Health and Human Development (USA)

The National Institutes for Health (USA)

**Appendix 2**

**Centre for Medical Statistics and Health Evaluation**

Shelley’s Cottage

Brownlow Street

Liverpool

L69 3GS

Telephone: 0151 794 5121

Facsimile: 0151 794 5130

Website: [www.liv.ac.uk/medstats/](http://www.liv.ac.uk/medstats/)

Email: [kerry.dwan@liv.ac.uk](mailto:kerry.dwan@liv.ac.uk)

Dear Sir/Madam,

I am involved in the MRC funded project ORBIT (Outcome Reporting Bias in Trials) (G0500952) investigating the degree and impact of outcome reporting bias (ORB) in Cochrane reviews, along with Professor Paula Williamson at the Centre for Medical Statistics and Health Evaluation, University of Liverpool, and Professor Doug Altman at the Centre of Statistics in Medicine, Oxford University.

As part of this project we are conducting a review of guidelines on research conduct from organisations that fund clinical trials.

We would be grateful if you could answer the following questions;

1. Do you fund clinical trials?

YES 

NO 

1. What is the maximum grant you have available?

…………………………………………………………………………………

…………………………………………………………………………………

1. Do you publish any guidelines for researchers conducting clinical trials?

YES 

NO 

1. Where can they be found? (e.g. web address)

…………………………………………………………………………………

…………………………………………………………………………………

1. How are they issued to researchers?

…………………………………………………………………………………

…………………………………………………………………………………

1. Are applicants referred to any other research guidelines?

…………………………………………………………………………………

…………………………………………………………………………………

1. Are applicants monitored in regards to the guidelines?

…………………………………………………………………………………

…………………………………………………………………………………

Your co-operation is much appreciated.

Yours sincerely

Kerry Dwan

PhD Student in Medical Statistics

**Appendix 3**

**Questionnaire**

1. Do you fund RCTs?

YES 

NO 

1. What is the maximum grant available?

…………………………………………………………………………………

…………………………………………………………………………………

1. Do you publish any guidelines for researchers conducting clinical trials?

YES  (If yes, go to question 4).

NO  (If no, email to ask questions 14, 15)

1. Where can they be found? (e.g. web address)[[4]](#footnote-5)

…………………………………………………………………………………

…………………………………………………………………………………

1. How are they issued to researchers?

…………………………………………………………………………………

…………………………………………………………………………………

1. Do the guidelines state that the trial has to be registered? If so, with which register?

…………………………………………………………………………………

…………………………………………………………………………………

1. In the guidelines, is there any reference to research conduct regarding the measuring and reporting of outcomes? If so, what is written and where can it be found?

…………………………………………………………………………………

…………………………………………………………………………………

1. In the guidelines, are there any references to the issues of protocol adherence/ changes to the protocol? If so, what is written and where can it be found?

…………………………………………………………………………………

…………………………………………………………………………………

1. In the guidelines, is there any reference to the declaration of outcomes as primary/secondary in the protocol? If so, is there any reference to the number of such outcomes? If so, what is written and where can it be found?

…………………………………………………………………………………

…………………………………………………………………………………

1. In the guidelines, is there any other specific reference to outcomes (i.e. not covered by question 7)? If so, what is written and where can it be found?

…………………………………………………………………………………

…………………………………………………………………………………

1. Do the guidelines state that the work should be published? If so, what, if any, recommendations are given regarding where should it be published?

…………………………………………………………………………………

…………………………………………………………………………………

1. Do the guidelines state what should be included in a publication (specifically regarding outcomes)?

…………………………………………………………………………………

…………………………………………………………………………………

1. Do their guidelines either use CONSORT as their guidelines or refer to them?

…………………………………………………………………………………

…………………………………………………………………………………

1. Are applicants referred to any other research guidelines?

…………………………………………………………………………………

…………………………………………………………………………………

1. Are applicants monitored with regards to adherence to the guidelines?

…………………………………………………………………………………

…………………………………………………………………………………

1. Recently listed charity [↑](#footnote-ref-2)
2. Now part of Epilepsy Research UK [↑](#footnote-ref-3)
3. No longer on AMRC website [↑](#footnote-ref-4)
4. Try to obtain guidelines and read through, answering questions 3 to 15. [↑](#footnote-ref-5)
