# Supplementary material for: Reporting of clinical trials: a review of research funders' guidelines
Source: Trials. 2008 Nov 25;9:66. doi: 10.1186/1745-6215-9-66 (PMC2630961; doi:10.1186/1745-6215-9-66)
Supplement: Additional file 2 — Table 3 Summary of organisation guidelines. A table of findings for organisations that issue guidelines [file 1745-6215-9-66-S2.doc]

**Table 3** Summary of Organisation guidelines

| **Organisation** | **Publish guidelines?** | **Do guidelines mention if a trial should be registered?** | **Do guidelines discuss protocol adherence?** | **Do guidelines state that work should be published? What guidance is given for the publication of results?** | **Are funders to be sent information on publications?** | **Are researchers monitored in regards to the guidelines?** | **Are researchers referred to other guidelines?** |
| --- | --- | --- | --- | --- | --- | --- | --- |
| Chief Scientist Office of the Scottish Executive Health Department | Y  Updated in 2006 | Y  This is the sponsors’ responsibility: ‘appropriate arrangements are in place for the registration of a trial.’ | Y^1^  Conducting a study to the agreed protocol (or proposal), in accordance with legal requirements, guidance and accepted standards of good practice. Substantive changes to the protocol or proposal are submitted for ethical review and for the sponsor’s agreement. These amendments are implemented only  when approved; | Y  CSO aims to ensure the widest and fastest possible dissemination of the research it supports; thereby encouraging the rapid uptake of research finding into clinical practice and attaches great importance to the publication and dissemination of the results of research undertaken with its grant support. | Y  A copy of the final, peer-reviewed version of all papers arising from the funded research and accepted for publication must be deposited in a publicly accessible repository (UK PubMed Central) and be made freely available within 6 months. | Y  Responsibility of organisation  ‘Ensuring studies are managed, monitored, and reported as agreed, according to the protocol.’ | MRC, ICH, Declaration of Helsinki,  Medicines for Human use (clinical trials) regulations 2004 (see MHRA) |
| Department of Health (England) | Y  Updated in 2005  (Also see HTA response) | Y  The Department of Health and the Medical Research Council (and soon the Wellcome Trust) have contracts with Current Controlled Trials to run trial registers on their behalf. Controlled trials are registered, and for clinical trials involving medicines, the research follows any conditions imposed by the licensing authority. | Y^1^  Conducting a study to the agreed protocol (or proposal), in accordance with legal requirements, guidance and accepted standards of good practice.  Substantive changes to the protocol or proposal are submitted for ethical review and for the sponsor’s agreement. These amendments are implemented only when approved. | Y  The findings from the work are opened to critical review through the accepted scientific and professional channels. | Y  Open access policy | Y  The Department of Health will work with its partners to develop coherent systems for monitoring research governance and addressing shortcomings. | MRC, ICH, Medicines for Human use (clinical trials) regulations 2004 (see MHRA) |
| Research and Development for Health and Social Care  (Wales)  (WORD Research funding scheme) | Y  Published in 2001, currently being updated | Y  Information is available on all research being undertaken in the organisation. This is held on a database, which contains details of funding, intellectual property rights, recruitment, research outputs and impact. It is important that where a clinical trials register exists that  studies are logged appropriately.  Principal investigator (PI) and sponsor role - Controlled trials are registered. | Y  Principal investigator role - Conducting research to the agreed protocol approved by the relevant ethics committee and the research sponsor. Any proposed changes or amendments to or deviations from the protocol are submitted for approval to the ethics committee, the research sponsor and any other appropriate body. | Y  It is the intention of both parties that the results of work carried out under the terms of this Agreement should be publishable. The “Institution” shall be permitted to present at conferences, symposia, or professional meetings, and to publish in journals, theses, or dissertations, or other media of their own choosing, the methods and results of research funded by the Grant | Y | N  Clinical trials in the UK are monitored by the MHRA through inspections. Any Clinical trials have to gain MHRA approval and ethical approval (via Research Ethics Committee administered by COREC). WORD does not monitor adherence to the Research Governance Framework (they are good practice guidelines). | MHRA, COREC, MRC Guidelines for Good Clinical Practice in Clinical Trials |
| World Health Organisation^2^ | Y (2002) | Y  Mentioned on website - International clinical trials registry platform [16] | Y^1^  A trial should be conducted in compliance with the protocol that has received prior institutional review board (IRB)/independent ethics committee (IEC) approval/favourable opinion. The investigator, or person designated by the investigator, should document and explain any deviation from the approved protocol. | Y^1^  Research funded by WHO is 1) peer reviewed by qualified global reviewers prior to publication and 2) subject to in-house review and approvals up to Assistant Director General or DG level.  In order to achieve its programmatic goals, WHO occasionally submits its copyright materials for publication by external publishers (e.g. articles submitted to peer-reviewed journals, chapters of books and books submitted for external publication on behalf of WHO). | open access policy | Y  Clinical studies are monitored for compliance to the guidelines and required regulations | CONSORT (mentioned in a bulletin in 2007), national regulation and guidelines, Guidelines for Good Clinical Practice (GCP) for Trials on Pharmaceutical Products, ICH, The document Good Clinical Practices: Document of the Americas |
| Food and Drug Administration (USA) | Y | Y | Y  Protocol amendments: This refers to protocol amendments but does not specifically refer to outcomes.  OOPD also stresses the importance of having an independent and unbiased/uninvolved party review the protocol and subsequent case report forms (CRFs) to ensure that the protocol is being adhered to. For more information, visit: http://www.fda.gov/orphan/. | Y | NS | Y  Monitoring is conducted via phone/email/written correspondence and/or in person by the Office of Orphan Product Development (OOPD) project officer to ensure that the investigator is making adequate progress, has adequate drug supply, and complies with other FDA requirements as described in our Request for Applications (RFA) and CFR. | ICH |
| Canadian Institutes of Health Research | 2007-2008 CIHR Grants and Awards Guide | NS | Y  Progress reports should indicate changes made to the protocol since approval, and that these changes have been approved by the responsible Research Ethics Board(s).  This is documented by having approval letters appended to progress reports. | Y  CIHR expects funded investigators to disseminate their research findings through peer-reviewed publications such as journals, manuscripts and books to maximize the impact and utility of their work. While investigators are free to choose the most appropriate vehicle, CIHR reminds those who publish in journals to consider one of the NRC Research Press journals, or other Canadian journals, among the options. | NS  CIHR does not explicitly state that publications be sent to us upon completion of the funded work. | Y  Progress reports are analyzed and investigators are informed of the results. Satisfactory reports are required to ensure continuation of funding. | CONSORT, ICH, Declaration of Helsinki |
| European Organisation for Research and Treatment of Cancer | Y  (2005) Internal working procedures are not made available externally. | Y  Trials are registered with the US National Cancer Institute PDQ (Physician data query) and a EudraCT number is obtained | Y  Amendments notified to relevant authorities - with respect to ICH GCP | Y  All trials are published unless they were prematurely closed to patient for poor patient accrual with very few patients having been entered. After revision by the Data Center and other co-authors (and the sponsor, if applicable) the manuscript will be sent out for publication to a major scientific journal. | Y | Y  Mainly by the EORTC Data Center and certain EORTC committees. | CONSORT, ICH, Declaration of Helsinki |
| Medical Research Council (MRC) (UK) | Y  (1998 and 2000, recently, clinical trials toolkit available on website) | Y  Registered at time of approval and publication with a current trial register. | Y^1^  Amendments reported to R&D and MHRA to be sent protocol. The REC needs to approve all substantial amendments.  A trial should be conducted in compliance with the protocol that has received prior Ethical committee favourable opinion and it is the responsibility of the principal investigator. | Y^1^  MRC subscribes to the guidance of the International Committee of Medical Journal Editors (see ICMJE, 1997)  The Principal Investigator should, subject to the procedures laid down by the Research Organisation, publish the results of the research in accordance with normal academic practice.  It is important to disseminate the results of clinical research, not only to the research community, but to the general public as well. The obvious route to inform the research community is through publication in scientific journals. It is recommended that the CONSORT guidelines are followed when preparing a manuscript for a clinical trial. This ensures that all relevant information about the trial is reported in the publication.  The PI should ensure that on completion of the study, the results are analysed, written up, reported and disseminated. TSC should ensure that appropriate efforts are made to ensure that the results of the trial are adequately disseminated and due consideration is given to the implementation of results in clinical practice. | Not a requirement; but the MRC has an Open access policy | Y  An MRC programme manager sits as an observer on each trial steering committee to monitor governance and progress. The MRC retain the right to undertake a clinical trial audit at any time.  This is the responsibility of the host institution and Trial steering committee | Department of Health Research Governance Framework, MHRA, COREC, CONSORT, Declaration of Helsinki. |
| Medical Research Council of South Africa (MRC-SA) | N | NA | NA | No  Although implicitly referred to through the Declaration of Helsinki. | No | NA | Declaration of Helsinki, Department of Health South Africa, ICH |
| The Health Technology Assessment Programme (UK) | Y | Y  All HTA funded RCTs are registered free of charge with the ISRCTN register. Non-RCTs are encouraged to register with clinicaltrials.gov to ensure publication. The ISRCTN register does accommodate non-RCTs. | NS  Although implicitly referred to through reference to MRC, Department of Health Research Governance Framework and The Medicines for Human Use (Clinical Trials) Regulations 2004. | Y  For any output (papers, abstracts, posters, press releases etc) from HTA research they encourage the widest dissemination in peer-review publications, at conferences etc. They encourage depositing any outputs into institutional repositories which most universities now use, in particular to support RAE returns | Y | Y  Carried out by a team at the National Co-ordinating Centre for HTA (NCCHTA). | MRC, Department of Health Research Governance Framework, The Medicines for Human Use (Clinical Trials) Regulations 2004, CONSORT |
| The National Cancer Institute (USA)^2^ | Y (2002) | Y  NCI has its own PDQ registry | Y  Any change to the approved protocol document must be documented point by point in a cover letter, and a replacement page(s) or a revised protocol document submitted to the protocol and information office | Y  The NIH Policy on Enhancing Public Access to Archived Publications Resulting from NIH-Funded Research (Public Access Policy) requests and strongly encourages all investigators to make their NIH-funded peer-reviewed, author's final manuscript available to other researchers and the public through the NIH National Library of Medicine's (NLM) PubMed Central (PMC) immediately after the final date of journal publication. | Y | Y  Monitoring occurs | NS |
| The National Health and Medical Research Council (Australia) | Y (Updated 2006) | Y  Refer to ICMJE [1]  The Australian Consumers’ Association (ACA) suggested that two national registers should be established, the one to provide a guide to all clinical  trials approved for conduct in Australia, and the other to provide a guide to results. | Y^1^  The trial should be conducted in compliance with the approved protocol. | Y^1^  To maximise the benefits from research, findings need to be disseminated as broadly as possible to allow access by other researchers and the wider  community. The NHMRC encourages researchers to consider the benefits of depositing their data and any publications arising from a research project in an appropriate subject and/or institutional repository wherever such a repository is available to the researcher(s). Any research outputs that have been or will be deposited in appropriate repositories should be identified in the Final Report. | NS | Y  Responsibility of institution | ICH, Declaration of Helsinki |
| The National Heart, Lung and Blood Institute (USA) | Y  (See NIH - Office for Extramural Research) | - | -  Although referred to implicitly through reference to ICH | Y  They have a voluntary Public Access Policy and will release a mandatory Public Access policy shortly (see <http://publicaccess.nih.gov/> (accessed September 2008)) | Y  List applicable publications in progress reports. | - | ICH |
| The National Institute for Allergies and Infectious Diseases (USA) | Y  (See NIH - Office for Extramural Research) | Y  It is the responsibility of the NIAID Division or Branch to register this trial in an acceptable registry.  <http://grants.nih.gov/grants/guide/notice-files/NOT-OD-08-023.html> (accessed September 2008) | Y  Plans for detecting, reviewing and reporting deviations from the protocol should be described. A statement may be included to indicate that deviations are not allowed, unless a statement is included in investigator agreement. Provisions for acknowledgement of deviations can be described. | Y  For all research (not just clinical trials), NIH has been encouraging researchers to submit their publications for public access in primary scientific journals. | Y | - | ICH, Declaration of Helsinki |
| The National Institute for Child Health and Human Development (USA) | Y  (See NIH -Office for Extramural Research) | - | - | - | - | - | - |
| The National Institutes for Health (USA)^2^ | Y  (See Office for Extramural Research – see NHLBI info) | - | - | - | - | - | - |
| Health and Social Care Research and Development Office (Northern Ireland) | Y (Updated 2006) | Y  Trials involving human participants must be registered, and for clinical trials involving medicines, the research follows any conditions imposed by the licensing authority | Y  Unless urgent safety measures are necessary, the research follows the protocol agreed by the relevant research ethics committee and by the sponsor, substantive changes to the protocol or proposal are submitted for ethical review and for the sponsor’s agreement. These amendments are implemented only when approved the findings from the work are opened to critical review through the accepted scientific and professional channels | Y^1^  Findings from research funded by the R&D Office should be disseminated but given the wide range of research they fund the method and type of dissemination will vary. | Y  Award holders provide an annual report which includes a list of publications and reports | Y  A monitoring service is available from the Clinical research Support Centre (CRSC) in NI. Trusts should decide the level of monitoring required for a trial and liaise with the CRSC to arrange for level of monitoring to be put in place | CONSORT, Medicines for human use (clinical trials) regulations 2004 (see MHRA), MRC |

1. Statement specific to ORB/ publication bias

2. Table not checked by organisation

Y Yes/Mentioned

No/ NS Not stated

NA Not Applicable

**References**

1. International Committee of Medical Journal Editors (ICMJE): **Uniform requirements for manuscripts submitted to biomedical journals.** *JAMA* 1997, **277**:927–934.
