# Supplementary material for: Reporting of clinical trials: a review of research funders' guidelines
Source: Trials. 2008 Nov 25;9:66. doi: 10.1186/1745-6215-9-66 (PMC2630961; doi:10.1186/1745-6215-9-66)
Supplement: Additional file 3 — Table 4 Summary of findings for charities that issue guidelines or terms and conditions. A table of findings for charities that issue guidelines [file 1745-6215-9-66-S3.doc]

Table 4 Summary of findings for charities that issue guidelines or terms and conditions

| **Charity** | **Do guidelines mention if a trial should be registered?** | **Do guidelines discuss protocol adherence?** | **Do guidelines state that work should be published? What guidance is given for the publication of results?** | **Are funders to be sent information on publications?** | **Are researchers monitored in regards to guidelines?** | **Are researchers referred to other guidelines?** |
| --- | --- | --- | --- | --- | --- | --- |
| Action Medical Research | Y  Grantholders will be expected to be included in the "The *meta*Register of Controlled Trails" database on [www.controlled-trials.com](http://www.controlled-trials.com/) (accessed September 2008) | NS  Although referred to implicitly through reference to AMRC guidelines. | Y  They would assume that the work will be publishable | NS | Y  It is the responsibility of the host institution. Once funding is in place it is up to the Universities and Hospitals to monitor the activity of its researchers. | AMRC |
| Action on Addiction | Y  Their clinical trials are registered - but this is done between researcher and university. | Y  Protocol adherence is discussed between researcher and their academic institution. | NS  Ideally they would like work to be published, but this is dependent on peer review, editor decision etc after submittal. (They don't provide guidance on publication as the researchers know the specialist journals they want to submit to). | Y | Y  Each researcher contract states that they will monitor progress. | University |
| Alzheimer's Research Trust | NS  Although referred to implicitly through reference to MRC guidelines. | NS  Although referred to implicitly through reference to MRC guidelines. | NS  Although referred to implicitly through reference to AMRC guidelines. | Y | Y  It is the responsibility of the host institution | MRC |
| Alzheimers society | NS | NS  Although referred to implicitly through reference to ICH guidelines. | Y  The Alzheimer’s Society attaches great importance to the dissemination of the results of research undertaken with grant support. Clinical intervention studies should include a formal Quality of Life outcome measure | Y | Y  All of their funded research is monitored regularly by their consumer monitors, and also by regular 6 monthly written reports | ICH, Declaration of Helsinki |
| Arthritis Research Campaign | NS  Although referred to implicitly through reference to AMRC guidelines. | NS  Although referred to implicitly through reference to AMRC guidelines. | Y  The host institution has a general duty to ensure that the useful results of the research are disseminated. It must accept responsibility for the proper evaluation of the research before it is published and, if the results are to be published in a reputable scientific or medical journal, it may rely on an evaluation of quality by the journal concerned. | Y  On acceptance for publication, a copy of the final manuscript of all peer reviewed research papers supported in whole or in part by an **arc** grant must be deposited in an open access archive such as PubMed Central (PMC) or UK PubMed Central (UKPMC), to be made freely available within six months of publication. | Y  It is the responsibility of the host institution. They request reports throughout the year, varying from progress reports before each Clinical Trial Collaboration Meeting and annual reports for the Arthritis Research Campaign from the PI's themselves. | AMRC, Institution’s Good Research Practice |
| Association for Spina Bifida and Hydrocephalus (ASBAH) | NS | Y  Any plans to modify or augment the objectives of the project, or any other major changes in approach, from those set out in the original grant application will require prior written agreement from ASBAH. Failure to secure such agreement may invalidate any contract between ASBAH and the host institution concerning financial support for the project. | NS | Y | NS | NS |
| Asthma UK | NS  Details of projects that receive financial support from Asthma UK are posted on the Asthma UK website.  Although referred to implicitly through reference to Department of Health Research Governance framework. | Y  Any significant deviations in research protocol from those stated in the grant application must be reported immediately to the Assistant Director, Research for Asthma UK to determine whether the grant may continue. | Y  Asthma UK encourages the publication of articles arising from the research that it supports and it is not its intention to interfere with such publications. | Y  Details of all studies and arising publications to be put on Asthma UK website. | Y  There are a number of monitoring stages during the lifetime of an Asthma UK funded grant/project. These include annual and final reports, both of which will be scrutinised by suitable independent experts in the field before they are approved and subsequent funding is agreed by Asthma UK.  They will be monitored against the Terms and Conditions of the award, and against any other prior knowledge about legislation or guidelines that either the reviewers or Asthma UK possess. | Department of Health Research Governance framework. |
| Ataxia UK | NS | NS | Y^1^  Ataxia UK requires the grant holder to submit the outcome of the research to peer- reviewed journals | Y  Ataxia UK should be sent copies of such papers at the time that they are submitted for publication. | NS | NS |
| Backcare | NS  Although referred to implicitly through reference to AMRC guidelines. | Y  Any major variation in the terms of a grant can only be considered on formal application to the Research Committee. | NS  Although referred to implicitly through reference to AMRC guidelines. | NS | NS  Although referred to implicitly through reference to AMRC guidelines. | AMRC, Institution, |
| Blackie Foundation Trust | NS  Although referred to implicitly through reference to AMRC guidelines. | NS  Although referred to implicitly through reference to AMRC guidelines. | Y  Must be published in a peer reviewed journal | Y | NS  Although referred to implicitly through reference to AMRC guidelines. | AMRC |
| Breakthrough Breast Cancer | NS  Although referred to implicitly through reference to MRC guidelines. | Y  Any plans to significantly change direction from the aims outlined in the original grant application will require the prior written agreement of Breakthrough. | Y  Before publication, Breakthrough requires that work undergoes the host institution’s normal procedures for ensuring the validity of the results and the suitability of the research for general publication.  Breakthrough requires its researchers to disseminate the results of the research that it funds in the usual manner, for example by publication and presentation at meetings. | Y | Y  It is the responsibility of the host institution and CRUK monitor too | Cancer Research UK, MRC, legal |
| Breast cancer campaign | NS | NS | NS | Y | NS | NS |
| British Heart Foundation | NS  Although referred to implicitly through reference to MRC guidelines. | NS  Although referred to implicitly through reference to MRC guidelines. | Y  The findings from the research funded by the Grant should be made freely available to the broader scientific community as soon as possible. | Y  BHF, as a member of the consortium of major UK biomedical and health research funders, contributes to the funding of UK PubMed Central (UKPMC), with the intention of ensuring that the complete versions of publications derived from their funded research are available freely. UKPMC was launched on 8 January 2007: see <http://www.ukpmc.ac.uk/funders/> (accessed September 2008). | Y  It is the responsibility of the host institution | Relevant guidelines issued nationally, MRC, Medicines for Human use (clinical trials) regulations 2004 (see MHRA) |
| British Lung Foundation | NS  Although referred to implicitly through reference to MRC and Wellcome Trust guidelines. | NS  Although referred to implicitly through reference to MRC guidelines. | NS  Although referred to implicitly through reference to MRC and Wellcome Trust guidelines. | Y | Y  It is the responsibility of the host institution.  BLF monitor via annual reports and site visits. | Wellcome Trust, MRC |
| British Retinitis Pigmentosa Society | Y  They would not fund unregistered work. | Y  The grant holders should highlight any difficulties that have arisen during the course of the study and modifications to the research protocol that have been required. | NS  Although referred to implicitly through reference to MRC guidelines. | Y | Y  Their researchers have to report to the satisfaction of our Medical Advisory Board and Board of Trustees, stage payments of a grant would be withheld until the standard of reporting was satisfactory. | MRC, AMRC, Retina International and the Genetic Interests Group |
| BUPA Foundation | NS  They have not previously demanded grantholders adhere to this as they have contributed to the National Research Register to assure such registration.  Although the situation has changed now that this registration has become a responsibility of the UKCRN and that they will need to include direction on this point.  Although referred to implicitly through reference to Wellcome Trust and Department of Health Research Governance Framework. | Y  They require any potential departure from protocol/plan to be notified in advance for approval by the Board. | Y  The Charities Act imparts a burden of responsibility on the Foundation’s Trustees to ensure that the results of any research supported by the Foundation are disseminated to the public at large. | Y  The Grant Holders are obliged to inform the Foundation, preferably in advance, of any publications or press coverage that may arise from their research project as well as presentations at any conferences | Y  It is the responsibility of the host institution. As a funder of projects, the Foundation is not in a position to apply the level of monitoring required, hence their application form requires applicants to identify the sponsor who will discharge this function. They do however keep in close touch with projects by regular communication and monitoring reports. If there is cause for concern, a monitoring visit will be made by Trustees. | AMRC, Wellcome Trust, Department of Health Research Governance Framework, research sponsors guidelines. |
| Cancer Research UK | Y | Y  Cancer Research UK understands that, in order to keep up with scientific  developments and publication of results arising from other laboratories, principal investigators may sometimes need to modify the aims and directions of their projects and these must be fully justified in Annual and Final reports. However, any plans to significantly change direction from the aims outlined in the original grant application will require the prior written agreement of Cancer Research UK. | Y  Cancer Research UK requires its researchers to promulgate the results of the research that it funds in the usual manner, for example by publication and presentation at meetings. | Y | Y  Applicants must produce an annual report which is reviewed before subsequent grants awarded makes sure they are adhering to terms and conditions/guidelines | MRC, Medicines for Human use (clinical trials) regulations 2004 (see MHRA) |
| Chest Heart And Stroke Scotland | NS | Y  If any significant changes occur in the project, or if the work deviates from the original proposal, CHSS must be informed immediately. | NS | Y | Y  On receipt of their application | NS |
| Children with Leukaemia | NS | NS | Y  The Grant Holder must make every effort to publicise their results, always ensuring that the Research is peer reviewed prior to it being published, publicised or disseminated. | Y  The Charity supports the open access approach which enables researchers to make their results available freely on the internet and retain copyright of the publication. The Grant Holder may apply to the Charity at the time of publication for support towards the additional costs of this. | NS | Leukaemia Research Fund |
| [Chronic Granulomatous Disorder Research Trust](http://www.cgd.org.uk/) | NS | NS  The clinical trial they fund is for gene therapy for very sick people – their very last treatment option therefore protocol adherence is meaningless in this context. | Y^1^  The Institution will ensure that publication of findings in peer reviewed journals is sought as soon as possible during, and after conclusion, of the Project even where results prove negative. | Y | NS | NS |
| CORE | NS  Although referred to implicitly through reference to MRC guidelines. | NS  Although referred to implicitly through reference to MRC guidelines. | Y  CORE expects that the results of research carried out during the period of its support will be published in peer-reviewed journal(s). | Y | NS  Although referred to implicitly through reference to MRC guidelines. | MRC |
| Cystic Fibrosis Trust | NS | NS | Y^1^  Grantholders are expected to seek publication of findings in peer reviewed journals as soon as possible during, and after conclusion, of the project even where results prove negative. | Y | Y  It is the responsibility of the host institution | NS |
| Diabetes Research and Wellness Foundation | NS  Although referred to implicitly through reference to AMRC guidelines. | NS  Although referred to implicitly through reference to AMRC guidelines. | NS  Although referred to implicitly through reference to AMRC guidelines. | Y | Y  DRWF Fellows send annual and final reports on the research they are conducting which are assessed by a member of our Research Advisory Board. | AMRC |
| Diabetes UK | NS  Although referred to implicitly through reference to MRC guidelines. | NS  Although referred to implicitly through reference to MRC guidelines. | Y  The charity requires that its grantholders disseminates the results of the research which it funds in the usual manner, for example, by publication and presentation at meetings | Y | Y  Applicants are monitored in regards to these guidelines by means of annual and final reports, which must be approved by Diabetes UK before the nest year's funding continues. | MRC |
| Epilepsy Research Foundation | NS  Although referred to implicitly through reference to Department of Health Research Governance Framework. | NS  Although referred to implicitly through reference to Department of Health Research Governance Framework. | NS  The Grantholder will assist the Charity in its policy of publicising as widely as possible its grants and progress in scientific research supported by the Charity. The grant holder is further encouraged to submit an abstract on an aspect of the work funded by Epilepsy Research UK to the ILAE UK Chapter Annual Scientific Meeting at least once during the lifetime of the grant. They are also encouraged to submit an abstract to their local Epilepsy Research UK-funded research network.  Although referred to implicitly through reference to Department of Health Research Governance Framework. | Y | NS  Although referred to implicitly through reference to Department of Health Research Governance Framework. | University, Department of Health Research Governance Framework |
| Foundation for the study of infant deaths^2^ | NS | NS | Y  A grantholder is expected to present and publish the results of research work undertaken subject to the normal practice of the institution | Y | Y  Grantholders are obliged to supply a brief description of their progress and any findings of general interest each year in August on forms provided. | NS |
| Guy's and St Thomas' Charity | NS  The charity only accepts applications via the R&D offices of the beneficiaries – these beneficiaries have their own rules, including registration of trials, so there is no need for the charity to stipulate this or any other governance requirement. | Y  Pre and post application, they state in their documentation that research projects must comply with the law and rules/regulations to which the grant is paid. | NS  Systematic dissemination process of publishing on their website the output of each grant they fund. Under their new strategy, they are putting the titles of publications on the web. | Y  A list of publications | Y  They have a monitoring and evaluation policy | Legal and Organisational |
| Heart Research UK | NS  Although referred to implicitly through reference to AMRC guidelines. | NS  Although referred to implicitly through reference to AMRC guidelines. | Y  Must be published | Y | NS  Although referred to implicitly through reference to AMRC guidelines. | AMRC |
| [Huntington's Disease Association](http://www.hda.org.uk/) | NS | NS | Y  They would expect work to be published or a report written | Y | NS | NS |
| Inspire Foundation (aka Integrated Spinal Rehabilitation Foundation) | NS  Although referred to implicitly through reference to Department of Health Research Governance Framework. | NS  Although referred to implicitly through reference to Department of Health Research Governance Framework. | Y  They assess whether plans are in place to disseminate the findings appropriately in accordance with the object of INSPIRE. Their objective is “to communicate the useful results of such research.” | Y | Y  It is the responsibility of the sponsor | Department of Health Research Governance Framework, AMRC, relevant legal, institutional or professional standards. |
| International Spinal Research Trust | Y  (refers to ICMJE) | NS  Although referred to implicitly through reference to ICH. | Y  At the conclusion of the project the Recipient shall arrange for all useful results thereof to be published, and may arrange for publication of interim results or other reports concerning the project at any time. All such publications shall appear in such journals and shall be presented in such manner as may be approved by ISRT. | Y | NS | Contributed to guidelines published in spinal cord journal [2-5], ICH |
| Juvenile Diabetes Research Foundation UK | NS  In US all trials must be registered. | Y  All JDRF funded grants must adhere to protocol submitted and if changes, we must approve. | Y | Y | Y  They conduct follow up reviews throughout the duration of the research / clinical study | Internal and Government guidelines. |
| Kidney Research UK | NS | NS | Y  In award terms and conditions. | Y | Y  According the protocol/objectives on the applications and thorough reporting on progress to the Charity | NS |
| Kids Kidney Research^2^ | NS  Although referred to implicitly through reference to AMRC guidelines. | NS  Although referred to implicitly through reference to AMRC guidelines. | NS  Although referred to implicitly through reference to AMRC guidelines. | Y | NS  Although referred to implicitly through reference to AMRC guidelines. | AMRC |
| Ludwig Institute for Cancer Research | Y  Clinical trials are registered on ClinicalTrials.gov, which is required by the ICMJE [1]. | Y  Proposed amendments of LICR sponsored protocols must be submitted to LICR’s Office of Clinical Trials Management for review and approval by the Protocol Review Committee, and then to the IRB (or HREC, as applicable). Amendments may be implemented only after a copy of the IRB’s approval letter has been transmitted to the Office of Clinical Trials Management at LICR. | Y  They expect all the research conducted by their investigators to be submitted for peer-review, and they do not restrict or limit the publication of any of their research. | NS | Y  Check the PIs are following international guidelines and the protocol.(ICH states it is sponsors responsibility) | ICH, Declaration of Helsinki, Applicable Laws and Regulations |
| Meningitis Research Foundation | NS  They remind the PI of the requirement to register the trial (refer to ICMJE [1]).  1  Although referred to implicitly through reference to MRC guidelines. | NS  Although referred to implicitly through reference to MRC guidelines. | Y  Disseminating and publicising research is essential to achieve the profile needed to attract support and reassure existing supporters that their money is being spent constructively. The Institution must ensure that all useful knowledge acquired from the Project research is disseminated to the public and others able to utilise or benefit from it. Where the research is highly technical, restricted access through medical publications, universities, and other medical and educational establishments to persons who have a sufficient reason to study the material will be acceptable. | Y | Y  The Principal Investigator on each project funded (whether trial or not) has to submit annual progress reports which are assessed by the scientific advisory panel, and continued funding is contingent on satisfactory progress | AMRC, Department of Health Research Governance Framework, Medicines for Human use (clinical trials) regulations 2004 (see MHRA), MRC Guidelines for Good Clinical Practice in Clinical Trials, other legal requirements or other applicable codes of best practice |
| Meningitis Trust | NS  Although referred to implicitly through reference to AMRC guidelines. | NS  Although referred to implicitly through reference to AMRC guidelines. | NS  They encourage publication.  Although referred to implicitly through reference to AMRC guidelines. | NS | NS  Although referred to implicitly through reference to AMRC guidelines. | AMRC |
| Meningitis UK | NS  Although referred to implicitly through reference to Department of Health’s Research Governance Framework. | Y  Any significant departures from the Project must be notified immediately in writing to MUK so that it may determine in its sole discretion whether to continue its support. | Y  MUK wishes (and is obliged as a matter of charity law) to ensure that the useful results of the Project research are published “for the public good”. | Y | Y  It is the responsibility of the host institution | Department of Health’s Research Governance Framework for Health and Social Care and the Medicines for Human Use (Clinical Trials) Regulations 2004. |
| [Motor Neurone Disease Association](http://www.mndassociation.org/) | NS | NS | Y  Grantees are expected to seek publication of findings in refereed journals during and as soon as possible after conclusion of the project | Y | NS | NS |
| Multiple Sclerosis Society of Great Britain and Northern Ireland | NS  Although referred to implicitly through reference to Department of Health’s Research Governance Framework and MRC guidelines. | Y  The Multiple Sclerosis Society understands that, in order to keep up with scientific developments and publication of results arising from other laboratories, the Principal Applicant may sometimes need to modify the aims and directions of their research and these must be fully justified in annual reports. However, any plans to significantly change direction from the aims outlined in the original grant application will require the prior written agreement of the Multiple Sclerosis Society. | Y^1^  Before publication, the Multiple Sclerosis Society requires that work undergoes the Host Institution’s normal procedures (involving external peer review) for ensuring the validity of the results and the suitability of the research for general publication.  The Multiple Sclerosis Society requires its researchers to disseminate the results of the research that it funds in the usual manner, for example by publication in peer reviewed journals and presentation at meetings.  Information is also disseminated regarding all the grants they fund in a lay format so that the outcomes of research are available in the public domain e.g. on the MS Society website | Y | Y  It is the responsibility of the host institution | University, Department of Health’s Research Governance Framework, MRC GCP |
| Muscular Dystrophy campaign^2^ | NS  Although referred to implicitly through reference to Department of Health’s Research Governance Framework. | Y  Any significant deviations in research protocol from those stated in the grant application must be reported immediately to the Head of Research of the MDC so that the MDC can determine whether the grant may proceed. | NS  Although referred to implicitly through reference to Department of Health’s Research Governance Framework. | Y | Y  It is the responsibility of the host institution | Department of Health’s Research Governance Framework, Medicines for Human Use (Clinical Trials) Regulations 2004, AMRC |
| National Eye Research Centre^2^ | NS  Although referred to implicitly through reference to Department of Health’s Research Governance Framework. | NS  Although referred to implicitly through reference to Department of Health’s Research Governance Framework and ICH. | NS  Although referred to implicitly through reference to Department of Health’s Research Governance Framework. | NS | NS  Although referred to implicitly through reference to Department of Health’s Research Governance Framework. | ICH, University and Department of Health Research Governance Framework |
| Novo Nordisk UK Research Foundation | NS  Although referred to implicitly through reference to Department of Health’s Research Governance Framework. | Y  Significant changes to awards will always be discussed by the board and the selection committees. | Y | NS | Y  Progress is monitored through annual reports to the foundation and targets for the coming year, also through an annual presentation to the selection committee and board of trustees. | Department of Health Research Governance Framework, ICH, Medicines for Human use (Clinical Trials) Regulations 2004 (see MHRA) |
| Parkinson's Disease Society of the UK | NS  Although referred to implicitly through reference to Department of Health’s Research Governance Framework. | Y  In terms of changing information in the project grant | Y  All PDS-funded projects will have details of their funding (project title, lay abstract/final report, name of grantholder, host institution, duration and value of support) placed in the public domain on the Society’s website. Publications and significant outcomes/outputs arising from the project will be added as they become available. | Y  Grantholders must abide by the Society’s policy on open access publishing. On acceptance for publication, a copy of the final manuscript of all peer reviewed research papers supported in whole or in part by a grant from PDS must be deposited in an open access archive such as PubMed Central or UK PubMed Central, to be made freely available within six months of publication. Any exceptions to this must be sanctioned by PDS. | Y  In terms of submitting reports | AMRC, Department of Health Research Governance Framework, Medicines for Human use (Clinical Trials) Regulations 2004 (see MHRA) |
| Remedi^2^ | NS  Although referred to implicitly through reference to AMRC guidelines | Y  The Director of Remedi must be notified immediately in writing or by email of any wish to revise the original protocol contained in the application which passed the Remedi peer review process and formed the basis upon which the award was made. The Principal Investigator cannot resubmit a revised protocol to the Ethics Committee of their respective institution, without the written approval of Remedi. | Y  Remedi, as a medical research charity, is under an obligation to ensure that the results of research which it funds in part or in whole are published for the public good. | Y | Y  Applicants are monitored we request a six monthly report and an annual report and we make an annual visit to the project. | AMRC, University or Institutions Guidelines for Good Practice. |
| Research into Ageing | NS  Although referred to implicitly through reference to Department of Health’s Research Governance Framework. | NS  Although referred to implicitly through reference to Department of Health’s Research Governance Framework. | Y^1^  Unless otherwise stated in the Offer Letter, Grantholders are required by the Charity to seek publication of findings in peer reviewed journals (as appropriate) as soon as possible during, and after conclusion, of the Project even where results prove negative. | Y | Y  It is the responsibility of the host institution | Department of Health Research Governance Framework, Institution, Medicines for Human use (clinical trials) regulations 2004 (see MHRA) |
| Samantha Dickson Brain Tumour Trust | NS  Any trial will need to be approved by the NCRI clinical brain tumour trials division. | Y  No significant change to the Project or its implementation in accordance with the details in the application form (as may be varied by the terms and conditions) may be made without the prior consent in writing of SDBTT. | Y | Y | Y  By organisation | NS |
| Sparks^2^ | NS | Y  Progress reports in the form specified by The Stroke Association must be submitted to the Research Department six months after the Start Date, and then annually in April of each year.  This is to ensure that the project is progressing in accordance with the information detailed in the original grant application. | NS | NS | Y  Progress reports in the form specified by The Stroke Association must be submitted to the Research Department six months after the Start Date, and then annually in April of each year.  This is to ensure that the project is progressing in accordance with the information detailed in the original grant application. | NS |
| The Stroke Association | NS  Although referred to implicitly through reference to Department of Health’s Research Governance Framework. | NS  Although referred to implicitly through reference to Department of Health’s Research Governance Framework. | Y  Work may be published subject to certain requirements | Y | NS  Although referred to implicitly through reference to Department of Health’s Research Governance Framework. | Department of Health Research Governance Framework |
| Saint Peter's Trust for Kidney, Bladder & Prostate Research^2^ | NS | NS | NS | Y | NS | NS |
| Sir Jules Thorn Charitable Trust (Terms and conditions not related to RCTs) | NS | NS | Y  Investigators are encouraged to submit their findings for publication in appropriate scientific journals | Y | Y | NS |
| Tommy’s The Baby Charity (confidential^2^) | NS  Although referred to implicitly through reference to MRC/ Wellcome Trust | NS  Although referred to implicitly through reference to MRC/ Wellcome Trust | NS  Although referred to implicitly through reference to MRC/ Wellcome Trust | NS | NS  Although referred to implicitly through reference to MRC/ Wellcome Trust | MRC/ Wellcome Trust |
| Tuberous Sclerosis Association^2^ | NS  Although referred to implicitly through reference to AMRC guidelines. | NS  Although referred to implicitly through reference to AMRC guidelines. | NS  Although referred to implicitly through reference to AMRC guidelines. | Y | NS  Although referred to implicitly through reference to AMRC guidelines. | AMRC |
| Wellbeing of woman | NS  Although referred to implicitly through reference to MRC guidelines and Department of Health Research Governance Framework. | NS  Although referred to implicitly through reference to MRC guidelines and Department of Health Research Governance Framework. | NS  It is strongly encouraged and referred to implicitly through reference to MRC guidelines and Department of Health Research Governance Framework. | Y | Y | AMRC, MRC, Department of Health Research Governance Framework |
| Wellchild | NS  Although referred to implicitly through reference to AMRC guidelines. | NS  Although referred to implicitly through reference to AMRC guidelines. | Y  The Charity intends that the results of the Project be published as widely as possible so that all elements of society might benefit directly or indirectly as a consequence of the findings. | Y | Y  It is the responsibility of the host institution  They meet with researchers every six months with annual written reports. This links with the payment schedule and helps them to ensure that the researchers are working within the guidelines. They also have a medical advisory board that can advice us on any specific issues that may arise. | AMRC |
| Wellcome trust (good research practice Guidelines updated 2005, clinical trials guidelines updated in 2007) | Y  Randomised controlled trials must be registered at the Wellcome Trust's clinical trials register <http://www.controlled-trials.com/mrct/> (accessed September 2008) to comply with International Committee of Medical Journal Editors [1] rules that it will consider a clinical trial for publication only if it has been registered in an appropriate registry. | NS  Although referred to implicitly through reference to MRC guidelines and Department of Health Research Governance Framework. | Y  Findings from research funded by the Grant should be published in an appropriate form, usually as papers in a refereed journal. | Y  Open access policy | Y  This is undertaken by the trial steering committees and data monitoring and ethics committees. The reports/minutes of those committees are expected to be fed back to the Wellcome Trust. | MRC, Department of Health Research Governance Framework, applicants undertaking clinical trials in developing countries are also referred to the Trust's position statement and guidance on the ethical principles relating to research involving people living in developing countries. |

1. Statement specific to ORB/ publication bias
2. Table not checked by charity

NS Not stated

Y Mentioned

**References**

1. International Committee of Medical Journal Editors (ICMJE): **Uniform requirements for manuscripts submitted to biomedical journals.** *JAMA* 1997, **277**:927–934.

2. Fawcett JW, Curt A, Steeves JD, Coleman WP, Tuszynski MH, Lammertse D, et al: **Guidelines for the conduct of clinical trials for spinal cord injury as developed by the ICCP panel: spontaneous recovery after spinal cord injury and statistical power needed for therapeutic clinical trials**. *Spinal Cord* 2007, **45**:190-205.

3. Lammertse D, Tuszynski MH, Steeves JD, Curt A, Fawcett JW, Rask C, et al: **Guidelines for the conduct of clinical trials for spinal cord injury as developed by the ICCP panel: clinical trial design**. *Spinal Cord* 2007, **45**:232-242

4. Steeves JD, Lammertse D, Curt A, Fawcett JW, Tuszynski MH, Ditunno JF, et al: **Guidelines for the conduct of clinical trials for spinal cord injury (SCI) as developed by the ICCP panel: clinical trial outcome measures**. *Spinal Cord* 2007, **45**:206-221.

5. Tuszynski MH, Steeves JD, Fawcett JW, Lammertse D, Kalichman M, Rask C, et al: **Guidelines for the conduct of clinical trials for spinal cord injury as developed by the ICCP Panel: clinical trial inclusion/exclusion criteria and ethics.** *Spinal Cord* 2007, **45**:222-231.
