# Supplementary material for: Reporting of clinical trials: a review of research funders' guidelines
Source: Trials. 2008 Nov 25;9:66. doi: 10.1186/1745-6215-9-66 (PMC2630961; doi:10.1186/1745-6215-9-66)
Supplement: Additional file 4 — Text box 1: Recommendations for text to include in research funders' guidelines. Recommendations for text to include in research funders' guidelines. [file 1745-6215-9-66-S4.doc]

**Text box 1: Recommendations for text to include in research funders’ guidelines**

- All pre-specified primary and secondary outcomes should be fully reported
- Any changes to the pre-specified outcomes from the protocol should be explained in the final report
- The choice of outcomes to be included in the final report should not be based on their results.
